# Antithrombin III treatment for portal vein thrombosis after living donor liver transplantation: a case report

**DOI:** 10.1186/s40792-020-00920-y

**Published:** 2020-07-03

**Authors:** Yuta Hirata, Yukihiro Sanada, Takahiko Omameuda, Takumi Katano, Go Miyahara, Naoya Yamada, Noriki Okada, Yasuharu Onishi, Yasunaru Sakuma, Naohiro Sata

**Affiliations:** Department of Surgery, Division of Gastroenterological, General and Transplant Surgery, Jichi Medical University, 3311-1 Yakushiji, Shimotsuke City, Tochigi 329-0498 Japan

**Keywords:** Antithrombin III, Portal vein thrombosis, Living donor liver transplantation, Liver transplantation

## Abstract

**Background:**

There have been no reports on the effectiveness of the administration of antithrombin III (AT III) for post-transplant portal vein thrombosis (PVT). We herein report a case of post-transplant PVT that was resolved by AT III treatment after living donor liver transplantation (LDLT).

**Case presentation:**

The patient was a 57-year-old man who had been diagnosed with decompensate liver cirrhosis by hepatitis C virus infection. He presented with repeated hepatic coma and refractory ascites. Computed tomography (CT) revealed PVT of Yerdel classification grade II before LDLT. He underwent ABO-identical LDLT using a right lobe graft. A liver function test revealed elevated liver enzyme levels on post-operative day (POD) 14. The CT examination on POD 15 revealed PVT in the left side of the main portal vein at the side of left gastric vein ligation. AT III treatment from POD 15 to POD 24 was performed. Magnetic resonance imaging revealed that the PVT had decreased 10% on POD 27. Furthermore, AT III treatment from POD 28 to POD 32 was performed. The CT examination demonstrated the disappearance of PVT on POD 69 and thereafter, he had no recurrence of PVT on 10 post-operative month (POM).

**Conclusions:**

The present case suggests that the administration of AT III is safe and suitable for the treatment of post-transplant PVT.

## Background

PVT is a serious and common problem in patients with liver cirrhosis who are candidates for liver transplantation (LT) [1]. PVT can significantly cause a poor recipient prognosis after LT [2] and can cause a contraindication of re-LT. Hidaka H et al. reported the effect of the administration of AT III for PVT in patients with chronic liver disease [3], and Imai H et al. reported the effect of the administration of AT III for pre-transplant PVT [4]. However, there have been no reports on the effectiveness of the administration of AT III for post-transplant PVT. We herein report a case of post-transplant PVT that was resolved by AT III treatment after LDLT.

## Case presentation

**Fig. 1:**
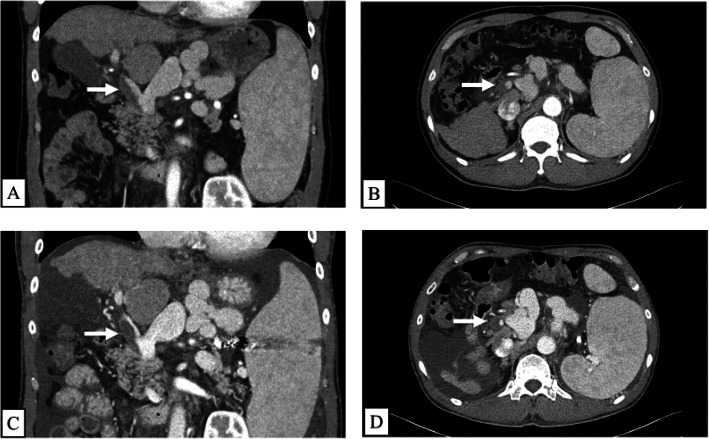
The CT findings of the pre-transplant PVT before and after the administration of AT III. The Yerdel classification of the pre-transplant PVT was grade II before the administration AT III (**a** and **b**). However, the pre-transplant PVT was progressed regardless of the administration AT III (**c** and **d**)

The patient was a 57-year-old man who had been diagnosed with decompensate liver cirrhosis by hepatitis C virus infection. He was treated with asunaprevir and daclatasvir from 2015 and achieved a sustained virological response. Since 2017, he presented with repeated hepatic coma and refractory ascites. His Child-Pugh score was 10 and model for end-stage liver disease score was 15. CT revealed PVT of Yerdel classification grade II before LDLT (Fig. 1a, b). AT III treatment (1500 units per day) for 10 days was performed twice but PVT was progressed (Fig. 1c, d). He underwent ABO-identical LDLT using a right lobe graft of his son. The graft weight was 545 g, and the graft-recipient body weight ratio was 0.81. For the recipient operation, Mercedes-Benz incisions were made. After dissection of the main portal vein (PV), the PVT was promptly removed and total hepatectomy was performed. The graft had five hepatic veins (HVs), including the right hepatic vein (RHV; 20 mm), the inferior RHV (IRHV; 9 mm), two V5 (7 and 10 mm), and V8 (12 mm) branches. The graft V5 and V8 branches were formed into single orifice using the recipient’s PV of the extracted liver and donor’s round ligament of the liver as anterior patch graft. Thereafter, HV reconstruction was performed between the recipient’s RHV and the graft formed HVs. IRHV was not anastomosed. The PV reconstruction was performed between the recipient’s main PV and the graft right PV. Hepatic artery reconstruction was performed between the recipient’s right hepatic artery and the graft right hepatic artery under a microscope. Biliary reconstruction was performed with a choledochocholedochostomy.

**Fig. 2:**
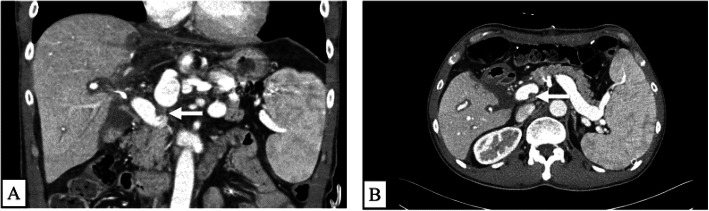
The CT findings of the post-transplant PVT before and the administration of AT III. Post-transplant PVT before the administration of AT III appeared in the left side of the main portal vein at the side of left gastric vein ligation (**a** and **b**)

**Fig. 3:**
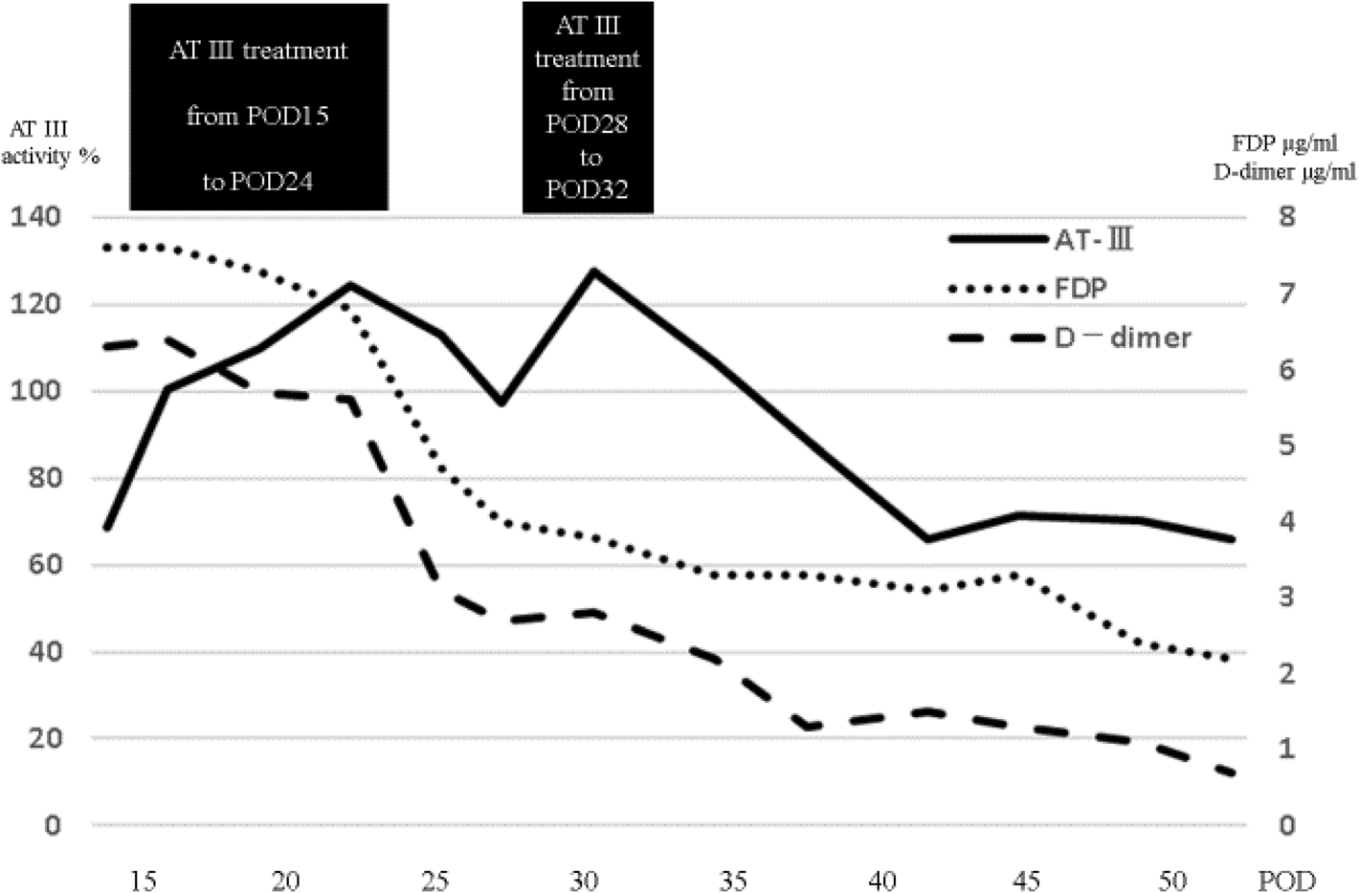
The chronological changes of AT III, FDP, D-dimer values period of the AT III treatment

**Fig. 4:**
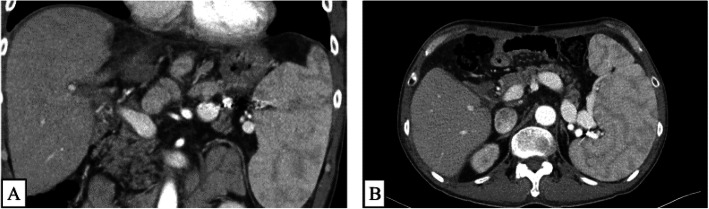
Post-transplant PVT was disappeared after the administration AT III (**a** and **b**)

Intra-operative and post-operative color Doppler ultrasonography was performed to assess the blood flow velocity and pattern. On POD 3, he was treated with low molecular weight heparin (LMWH) as prophylaxis against post-operative venous thrombosis. On POD 5, anticoagulant therapy was ceased due to abdominal bleeding. The abdominal bleeding improved by conservative treatment. Thereafter, the post-operative course was good; however, a liver function test revealed elevated liver enzyme levels on POD 14. The laboratory findings on POD 14 were as follows: total bilirubin, 2.76 mg/dl; direct bilirubin, 0.49 mg/dl; aspartate amino transferase, 40 U/L; alanine aminotransferase, 122 U/L; prothrombin time, 14.1 s; activated partial thromboplastin time, 37.5 s; AT III activity, 68.7 %; fibrinogen degradation product (FDP), 7.6 μg/ml; D-dimer, 6.3 μg/ml; protein C, 71.9%; platelet 5.0 × 10^4^/μℓ. The periodic CT examination on POD 15 revealed PVT in the left side of the main PV at the side of left gastric vein ligation (Fig. 2a, b). AT III treatment (1500 units per day) from POD 15 to POD 24 was performed. Magnetic resonance imaging revealed that the PVT had decreased 10% on POD 27. Furthermore, AT III treatment (1500 units per day) from POD 28 to POD 32 was performed. Figure 3 showed the chronological changes of AT III, FDP, D-dimer during the period of AT III treatment. During the period of AT III treatment, the AT III activity remained above 100%, and FDP and D-dimer levels decreased. On POD 52, he was discharged from the hospital. The CT examination demonstrated the disappearance of PVT on POD 69 (Fig. 4a, b) and thereafter, he had no recurrence of PVT on 10 POM.

## Discussion

PVT occurs in approximately 3-7% after LT and can be fatal for both the graft and recipient [5–8]. Factors associated with post-transplant PVT include technical issues, preexisting PVT requiring thromboendovenectomy at the time of LT, small PV size (< 5 mm), earlier splenectomy, and use of venous conduits for PV reconstruction [5–8]. In this case, he had preexisting PVT requiring thromboendovenectomy at the time of LT and he had risk factor for post-transplant PVT. At present, recipients with post-transplant PVT have been treated with anticoagulation (heparin or LMWH or vitamin K antagonists and so on) [9–11]. But there are no standard treatments for post-transplant PVT.

AT III is a coagulation regulator synthesized in the liver. AT III is a physiologic inhibitor of thrombin, factor Xa, and other serine proteases. The administration of AT III suppresses the hypercoagulable state, and PVT disappears due to the secondary action of the fibrinolytic system. Hidaka H et al. reported the effect of the administration of AT III for PVT in patients with chronic liver disease [3]. However, there have been no reports about the usefulness of AT III treatment for post-transplant PVT. The present case was only treated with AT III for PVT, and PVT disappeared. Therefore, PVT was dissolved with AT III alone in the present case. The patient showed no complications after the administration of AT III and was discharged. Hidaka H et al. reported the overall incidences of adverse effects did not differ significantly between the AT III group and the placebo group [3]. The most important advantages of AT III over anticoagulants are expected to include fewer adverse effects and an extremely low likelihood of serious life-threatening complications, especially bleeding [3]. As of 10 POM, he had no signs of recurrence of PVT.

## Conclusions

Although the accumulation of experience of similar cases will be necessary to validate these findings, the present case suggests that the administration of AT III is safe and suitable for the treatment of post-transplant PVT.

## Data Availability

We confirm that the data supporting the findings of this study are available within the article and its supplementary materials.

## Abbreviations

AT III: Antithrombin III
PVT: Portal vein thrombosis
LDLT: Living donor liver transplantation
CT: Computed tomography
POD: Post-operative day
LT: Liver transplantation
PV: Portal vein
HVs: Hepatic veins
RHV: Right hepatic vein
IRHV: Inferior right hepatic vein
LMWH: Low molecular weight heparin
FDP: Fibrinogen degradation product
POM: Post-operative month
